# Supplementary material for: A Likelihood Approach for Real-Time Calibration of Stochastic Compartmental Epidemic Models
Source: PLoS Comput Biol. 2017 Jan 17;13(1):e1005257. doi: 10.1371/journal.pcbi.1005257 (PMC5240920; doi:10.1371/journal.pcbi.1005257)
Supplement: S1 File — (TAR.GZ) [file pcbi.1005257.s014.tar.gz › HSPH_Online-SI-Revision/IPoi09/n100-severe/n100-severe_BENCH2_table-4.pdf]

1	newly 3 weeks spec	6483.	7849.	{5054., 9307.}	30.9143	0.129956	newly 3 weeks cumul	16 417.	22 585.5	{12 051., 27 019.}	48.8384	0.190006	newly 5 weeks spec	6768.	6583.57	{4602., 9245.}	23.2533	0.108561	newly 5 weeks cumul	30 019.	36 575.2	{26 202., 41 648.}	29.0828	0.119089	newly 54 weeks cumul 56 585. 59 022.8 {47 221., 73 130.} 15.4297 0.0661515
2	newly 3 weeks spec	9252.	11 434.3	{7184., 14 501.}	31.6518	0.120592	newly 3 weeks cumul	20 576.	33 354.5	{19 032., 38 758.}	67.3679	0.224779	newly 5 weeks spec	7700.	6158.08	{2412., 11 296.}	44.4282	0.252455	newly 5 weeks cumul	37 668.	48 595.9	{37 989., 54 406.}	29.9248	0.111546	newly 61 weeks cumul 50 118. 56 453.1 {45 260., 65 808.} 18.0785 0.072306
3	newly 3 weeks spec	13 618.	14 714.2	{5121., 18 224.}	31.775	0.168996	newly 3 weeks cumul	28 056.	34 656.8	{7419., 48 304.}	52.7286	0.254162	newly 5 weeks spec	10 200.	11 230.4	{7080., 17 553.}	31.305	0.127461	newly 5 weeks cumul	51 910.	59 706.2	{37 148., 67 852.}	27.4162	0.118702	newly 62 weeks cumul 64 948. 80 712.4 {75 538., 85 619.} 24.2723 0.0936595
4	newly 3 weeks spec	8496.	6710.52	{4151., 11 283.}	33.6295	0.184861	newly 3 weeks cumul	19 445.	25 994.7	{18 270., 32 552.}	39.7392	0.145637	newly 5 weeks spec	8732.	2743.26	{1151., 5161.}	70.6955	0.611525	newly 5 weeks cumul	37 352.	33 149.1	{24 606., 45 653.}	20.8955	0.104276	newly 60 weeks cumul 61 359. 36 690.2 {25 880., 51 809.} 40.2041 0.242554
5	newly 3 weeks spec	5838.	5692.21	{2964., 8932.}	28.5651	0.134636	newly 3 weeks cumul	15 689.	19 923.1	{12 963., 25 688.}	34.5628	0.129614	newly 5 weeks spec	5980.	3384.89	{1310., 7139.}	51.1521	0.350008	newly 5 weeks cumul	27 938.	27 787.9	{16 541., 39 251.}	22.6278	0.104987	newly 54 weeks cumul 56 535. 35 369.7 {18 933., 56 955.} 38.6582 0.239688
6	newly 3 weeks spec	8007.	5102.57	{2708., 8833.}	42.0589	0.2743	newly 3 weeks cumul	17 018.	20 631.5	{14 653., 27 671.}	33.0394	0.139093	newly 5 weeks spec	9118.	2085.43	{808., 4778.}	77.1284	0.753524	newly 5 weeks cumul	35 492.	26 056.1	{16 769., 38 416.}	30.0498	0.17288	newly 60 weeks cumul 63 071. 29 787.1 {18 492., 44 701.} 52.7721 0.353677
7	newly 3 weeks spec	9834.	10 028.	{4287., 12 870.}	25.6534	0.132899	newly 3 weeks cumul	19 745.	27 478.5	{6835., 35 539.}	62.024	0.251999	newly 5 weeks spec	11 408.	7038.57	{3196., 12 523.}	42.2603	0.28161	newly 5 weeks cumul	43 078.	43 311.9	{27 301., 49 711.}	15.6532	0.0772119	newly 61 weeks cumul 66 246. 56 274.4 {47 010., 64 696.} 15.0523 0.074731
8	newly 3 weeks spec	6637.	7558.82	{4933., 9451.}	25.847	0.104918	newly 3 weeks cumul	16 097.	21 544.9	{15 006., 26 566.}	38.6636	0.138937	newly 5 weeks spec	7091.	5739.	{2022., 9231.}	35.4544	0.213123	newly 5 weeks cumul	30 302.	34 195.1	{25 866., 39 673.}	19.4668	0.0784008	newly 57 weeks cumul 53 290. 46 608.3 {28 987., 58 427.} 17.4194 0.0916719
9	newly 3 weeks spec	10 162.	7008.22	{1939., 11 889.}	39.0329	0.278604	newly 3 weeks cumul	16 274.	12 392.4	{2926., 22 193.}	43.5626	0.293411	newly 5 weeks spec	13 814.	13 175.7	{9127., 15 126.}	12.789	0.0729532	newly 5 weeks cumul	44 791.	36 358.7	{16 720., 51 543.}	26.0332	0.161988	newly 62 weeks cumul 61 547. 89 909.8 {74 583., 92 966.} 46.0831 0.163451
10	newly 3 weeks spec	8608.	5352.79	{1225., 9019.}	39.7434	0.341911	newly 3 weeks cumul	19 464.	12 018.4	{2309., 22 399.}	44.3932	0.384627	newly 5 weeks spec	8668.	7529.95	{3394., 9395.}	19.5757	0.148855	newly 5 weeks cumul	37 387.	26 170.7	{7740., 40 726.}	33.5984	0.268903	newly 55 weeks cumul 57 915. 87 513.8 {82 172., 91 433.} 51.1073 0.178911
11	newly 3 weeks spec	9885.	12 365.2	{9668., 14 686.}	35.7016	0.161524	newly 3 weeks cumul	20 715.	29 381.5	{16 457., 40 129.}	55.8796	0.226108	newly 5 weeks spec	9915.	9452.84	{4828., 13 375.}	27.1858	0.136796	newly 5 weeks cumul	41 751.	50 693.9	{44 999., 56 366.}	28.5823	0.124224	newly 61 weeks cumul 59 863. 67 691.1 {59 516., 72 957.} 14.1308 0.0568582
12	newly 3 weeks spec	6820.	7356.27	{3185., 9563.}	32.5836	0.171256	newly 3 weeks cumul	17 074.	18 599.7	{6151., 27 581.}	42.5623	0.221811	newly 5 weeks spec	6925.	7986.27	{6018., 9517.}	24.7769	0.109641	newly 5 weeks cumul	31 324.	34 516.	{18 043., 43 142.}	29.7684	0.147772	newly 54 weeks cumul 59 414. 75 186.4 {62 405., 83 056.} 27.0062 0.101689
13	newly 3 weeks spec	6696.	7245.26	{4430., 9342.}	23.0347	0.0975223	newly 3 weeks cumul	17 080.	23 618.1	{18 108., 27 331.}	41.2829	0.14949	newly 5 weeks spec	6771.	4825.52	{2270., 8244.}	37.6472	0.222655	newly 5 weeks cumul	30 784.	34 526.5	{23 707., 41 402.}	21.0112	0.0856465	newly 54 weeks cumul 57 756. 47 021.9 {28 285., 65 517.} 24.3463 0.129515
14	newly 3 weeks spec	5021.	5758.	{3268., 8798.}	34.5046	0.136062	newly 3 weeks cumul	12 730.	19 853.7	{14 639., 24 490.}	56.8768	0.188226	newly 5 weeks spec	5391.	3579.37	{1527., 7444.}	48.1036	0.302779	newly 5 weeks cumul	23 614.	28 105.1	{18 257., 38 889.}	30.4576	0.116041	newly 54 weeks cumul 54 929. 36 216.1 {21 226., 58 366.} 36.1436 0.218785
15	newly 3 weeks spec	6167.	8683.69	{7378., 10 007.}	48.4417	0.211191	newly 3 weeks cumul	14 161.	24 260.8	{15 830., 29 002.}	79.581	0.294944	newly 5 weeks spec	6966.	7253.53	{4735., 9994.}	26.8259	0.138953	newly 5 weeks cumul	28 290.	39 774.4	{35 519., 44 232.}	47.7394	0.205194	newly 56 weeks cumul 48 068. 63 824.8 {50 450., 75 039.} 33.1078 0.120039
16	newly 3 weeks spec	5400.	7544.53	{3835., 9408.}	53.2539	0.216935	newly 3 weeks cumul	13 040.	21 158.7	{7862., 27 009.}	78.86	0.290704	newly 5 weeks spec	6295.	6689.68	{3809., 9144.}	26.9501	0.125099	newly 5 weeks cumul	25 735.	35 149.4	{21 279., 41 865.}	47.6231	0.192111	newly 55 weeks cumul 58 209. 61 653.4 {45 677., 75 513.} 16.3647 0.0697657
17	newly 3 weeks spec	7444.	7136.32	{3593., 9978.}	28.9367	0.185192	newly 3 weeks cumul	14 892.	22 223.9	{16 577., 27 215.}	58.1011	0.25291	newly 5 weeks spec	8949.	3512.5	{934., 6618.}	60.7498	0.516435	newly 5 weeks cumul	32 852.	31 210.5	{21 693., 37 975.}	18.0313	0.12681	newly 61 weeks cumul 50 701. 37 327.4 {26 865., 46 342.} 26.3775 0.145177
18	newly 3 weeks spec	7109.	6316.31	{3277., 10 005.}	28.8508	0.147586	newly 3 weeks cumul	17 024.	23 699.5	{17 003., 27 653.}	40.1666	0.141495	newly 5 weeks spec	7086.	2762.12	{895., 7224.}	62.8504	0.515221	newly 5 weeks cumul	31 968.	30 825.7	{19 765., 40 520.}	18.4073	0.0865803	newly 59 weeks cumul 50 495. 34 428.6 {20 858., 51 404.} 32.6115 0.188133
19	newly 3 weeks spec	9821.	11 640.8	{4443., 14 172.}	37.126	0.18338	newly 3 weeks cumul	23 696.	28 124.6	{7186., 38 928.}	44.6728	0.226464	newly 5 weeks spec	7954.	10 643.3	{7553., 13 685.}	39.2523	0.14474	newly 5 weeks cumul	41 504.	50 585.2	{28 112., 58 772.}	34.9866	0.154879	newly 59 weeks cumul 56 218. 82 369.3 {76 324., 89 040.} 46.5177 0.16469
20	newly 3 weeks spec	8828.	7912.24	{2616., 11 019.}	27.0318	0.19163	newly 3 weeks cumul	20 393.	17 899.4	{4589., 28 096.}	34.8422	0.242248	newly 5 weeks spec	8356.	9657.59	{7344., 11 110.}	28.2432	0.129467	newly 5 weeks cumul	37 963.	36 795.5	{16 447., 48 538.}	24.2081	0.149938	newly 57 weeks cumul 56 638. 88 180.3 {82 981., 92 721.} 55.6911 0.191809
21	newly 3 weeks spec	9444.	8794.92	{5315., 12 771.}	23.9242	0.120201	newly 3 weeks cumul	18 625.	28 589.3	{14 145., 35 157.}	62.2903	0.215717	newly 5 weeks spec	10 527.	5117.46	{1795., 12 206.}	56.9627	0.447185	newly 5 weeks cumul	40 402.	40 745.4	{30 744., 50 045.}	14.7	0.0670526	newly 61 weeks cumul 60 507. 48 590.4 {34 506., 63 786.} 21.7087 0.116611
22	newly 3 weeks spec	7683.	7464.73	{5232., 9293.}	17.665	0.082272	newly 3 weeks cumul	14 543.	21 411.6	{10 077., 25 557.}	57.7448	0.203255	newly 5 weeks spec	9422.	5575.9	{2731., 9286.}	41.3235	0.273587	newly 5 weeks cumul	33 493.	33 770.7	{27 305., 38 966.}	11.6241	0.0517837	newly 59 weeks cumul 53 168. 46 037.7 {34 941., 57 375.} 16.7361 0.0839299
23	newly 3 weeks spec	16 661.	9479.38	{4392., 12 161.}	43.1043	0.299905	newly 3 weeks cumul	28 675.	26 068.6	{6761., 33 098.}	21.9571	0.175509	newly 5 weeks spec	10 882.	5035.81	{1666., 11 669.}	56.9575	0.429612	newly 5 weeks cumul	56 693.	38 461.5	{28 925., 43 538.}	32.1584	0.195305	newly 63 weeks cumul 64 940. 45 814.3 {39 809., 50 938.} 29.4513 0.153221
24	newly 3 weeks spec	11 463.	11 325.9	{6897., 14 949.}	19.2878	0.090093	newly 3 weeks cumul	20 345.	32 954.9	{20 465., 37 308.}	65.7319	0.217128	newly 5 weeks spec	12 907.	3458.37	{893., 8394.}	73.2055	0.694151	newly 5 weeks cumul	47 801.	43 520.6	{38 170., 47 106.}	8.95456	0.0418048	newly 64 weeks cumul 66 552. 45 675.8 {38 873., 50 744.} 31.3682 0.165166
25	newly 3 weeks spec	11 140.	15 575.7	{7237., 18 523.}	53.5336	0.301077	newly 3 weeks cumul	19 297.	34 392.4	{10 241., 44 789.}	92.9448	0.406815	newly 5 weeks spec	11 133.	7678.84	{3696., 12 922.}	42.3663	0.35031	newly 5 weeks cumul	44 204.	54 591.	{42 846., 59 554.}	30.0122	0.231403	newly 64 weeks cumul 57 475. 61 601.3 {56 905., 66 173.} 12.97 0.172369
26	newly 3 weeks spec	9973.	8412.77	{4450., 11 898.}	27.285	0.201744	newly 3 weeks cumul	21 090.	18 544.5	{7887., 30 310.}	37.4119	0.254893	newly 5 weeks spec	10 139.	10 453.3	{7973., 11 964.}	16.8169	0.0958518	newly 5 weeks cumul	42 295.	38 965.4	{25 374., 52 143.}	22.9632	0.150515	newly 58 weeks cumul 65 347. 87 906.5 {81 698., 91 342.} 34.5226 0.128363
27	newly 3 weeks spec	9435.	8817.35	{2384., 12 826.}	32.9436	0.221746	newly 3 weeks cumul	19 821.	18 981.	{4053., 30 761.}	42.3326	0.272412	newly 5 weeks spec	9500.	10 916.4	{7386., 12 892.}	26.9798	0.123987	newly 5 weeks cumul	40 049.	40 392.3	{16 584., 53 878.}	28.4226	0.164675	newly 59 weeks cumul 58 495. 85 842.9 {81 150., 90 426.} 46.7526 0.166088
28	newly 3 weeks spec	6339.	8035.69	{5286., 11 128.}	37.5221	0.135598	newly 3 weeks cumul	16 315.	27 763.4	{21 466., 32 618.}	71.5744	0.230084	newly 5 weeks spec	5894.	4099.81	{1824., 7089.}	45.9521	0.282748	newly 5 weeks cumul	28 643.	37 834.8	{28 981., 46 556.}	34.5891	0.123834	newly 58 weeks cumul 47 081. 44 250.1 {31 356., 58 738.} 21.692 0.102865
29	newly 3 weeks spec	9036.	10 968.5	{8406., 12 871.}	28.9108	0.128786	newly 3 weeks cumul	21 261.	31 981.9	{29 021., 35 515.}	55.6028	0.213237	newly 5 weeks spec	8506.	6418.55	{3234., 8871.}	31.129	0.179923	newly 5 weeks cumul	39 366.	47 315.7	{41 290., 51 761.}	24.7321	0.106749	newly 59 weeks cumul 60 373. 59 352.3 {46 233., 70 495.} 12.2756 0.0550854
30	newly 3 weeks spec	6346.	7292.38	{3551., 9772.}	37.0397	0.196118	newly 3 weeks cumul	15 783.	17 075.7	{6810., 25 124.}	38.9803	0.218936	newly 5 weeks spec	6273.	8771.67	{7634., 9837.}	48.7076	0.194853	newly 5 weeks cumul	28 864.	34 224.7	{19 760., 43 909.}	35.8692	0.175924	newly 55 weeks cumul 48 852. 86 537.1 {81 421., 91 474.} 77.1414 0.247775
31	newly 3 weeks spec	15 806.	15 375.3	{9066., 18 393.}	14.711	0.0771596	newly 3 weeks cumul	27 617.	33 688.8	{21 684., 42 259.}	35.3122	0.15001	newly 5 weeks spec	9080.	7524.38	{3816., 12 922.}	37.6731	0.203997	newly 5 weeks cumul	52 596.	53 772.7	{45 244., 59 437.}	8.57892	0.0376208	newly 64 weeks cumul 59 587. 60 313.7 {54 324., 65 873.} 7.72072 0.0336755
32	newly 3 weeks spec	10 271.	12 474.4	{6878., 16 311.}	39.3801	0.15909	newly 3 weeks cumul	19 060.	25 488.2	{10 955., 40 304.}	57.9992	0.217873	newly 5 weeks spec	11 191.	13 107.5	{9069., 16 248.}	26.6907	0.105184	newly 5 weeks cumul	42 799.	52 858.7	{39 723., 62 002.}	28.7882	0.109931	newly 62 weeks cumul 60 379. 78 911.5 {75 053., 81 187.} 30.6937 0.115734
33	newly 3 weeks spec	12 313.	10 620.	{6123., 14 678.}	23.911	0.125678	newly 3 weeks cumul	26 701.	33 431.	{29 421., 38 230.}	32.1115	0.130593	newly 5 weeks spec	10 094.	4418.39	{1540., 8056.}	60.3853	0.476291	newly 5 weeks cumul	49 375.	45 270.6	{36 310., 51 432.}	10.2214	0.0494582	newly 62 weeks cumul 64 967. 49 747.1 {38 988., 58 333.} 23.4271 0.121039
34	newly 3 weeks spec	7987.	6553.33	{1981., 9348.}	28.9717	0.247113	newly 3 weeks cumul	19 140.	16 043.	{3786., 26 043.}	38.1539	0.300386	newly 5 weeks spec	7767.	7732.2	{4808., 9460.}	17.9087	0.135481	newly 5 weeks cumul	35 440.	31 119.8	{11 689., 42 522.}	26.1576	0.207009	newly 54 weeks cumul 57 950. 80 476.3 {70 989., 86 915.} 38.872 0.141084
35	newly 3 weeks spec	12 123.	9398.85	{2400., 13 150.}	26.4399	0.210018	newly 3 weeks cumul	20 957.	20 759.7	{3559., 34 557.}	45.2502	0.282342	newly 5 weeks spec	11 360.	8269.73	{3285., 12 616.}	31.0278	0.200021	newly 5 weeks cumul	47 195.	38 803.5	{19 472., 46 966.}	17.876	0.122487	newly 63 weeks cumul 60 555. 53 912.5 {48 177., 57 058.} 10.9694 0.0512629
36	newly 3 weeks spec	8896.	8026.08	{2330., 12 946.}	38.9477	0.252094	newly 3 weeks cumul	16 239.	15 124.1	{3683., 26 399.}	47.1911	0.293517	newly 5 weeks spec	12 338.	12 562.4	{9341., 14 614.}	17.039	0.0916909	newly 5 weeks cumul	40 963.	38 743.	{17 701., 54 058.}	27.7153	0.163638	newly 61 weeks cumul 64 577. 90 833.7 {88 139., 93 572.} 40.6595 0.148047
37	newly 3 weeks spec	11 908.	10 029.3	{7275., 12 752.}	20.3104	0.109425	newly 3 weeks cumul	22 407.	29 873.3	{20 209., 35 186.}	42.3444	0.165269	newly 5 weeks spec	9251.	4539.44	{1416., 9965.}	57.1921	0.451535	newly 5 weeks cumul	44 615.	41 885.4	{37 975., 44 935.}	7.1714	0.0343505	newly 63 weeks cumul 54 448. 46 204. {39 536., 53 809.} 15.141 0.0742858
38	newly 3 weeks spec	11 993.	11 592.8	{4193., 15 522.}	27.1741	0.160011	newly 3 weeks cumul	24 507.	24 822.5	{6657., 40 834.}	40.2084	0.222568	newly 5 weeks spec	11 210.	12 328.5	{7492., 15 104.}	22.9808	0.0971915	newly 5 weeks cumul	48 530.	50 197.7	{29 093., 60 974.}	21.6534	0.106121	newly 61 weeks cumul 66 756. 80 998.3 {74 009., 84 703.} 21.3348 0.0833982
39	newly 3 weeks spec	6786.	3947.38	{1910., 7837.}	48.8245	0.326765	newly 3 weeks cumul	16 864.	16 741.	{10 705., 25 199.}	25.8023	0.119721	newly 5 weeks spec	6041.	1767.5	{628., 3787.}	76.2323	0.722721	newly 5 weeks cumul	29 945.	21 173.1	{12 407., 35 076.}	35.9549	0.213416	newly 58 weeks cumul 46 740. 23 856.3 {13 173., 41 302.} 51.086 0.349559
40	newly 3 weeks spec	7110.	5636.65	{2061., 8754.}	36.1906	0.210075	newly 3 weeks cumul	16 261.	19 512.8	{8322., 24 712.}	33.4543	0.136697	newly 5 weeks spec	7170.	2856.3	{441., 5774.}	68.0642	0.6071	newly 5 weeks cumul	31 380.	26 516.7	{15 895., 33 951.}	20.1552	0.106263	newly 61 weeks cumul 47 800. 30 004.3 {16 132., 41 559.} 37.2295 0.222997
41	newly 3 weeks spec	12 763.	10 626.5	{4907., 15 467.}	28.8349	0.158242	newly 3 weeks cumul	25 803.	37 846.7	{31 847., 41 250.}	46.6756	0.163806	newly 5 weeks spec	9905.	2462.39	{777., 4714.}	75.14	0.67892	newly 5 weeks cumul	48 570.	45 940.7	{34 447., 52 233.}	10.2925	0.0497052	newly 63 weeks cumul 62 184. 47 576.4 {34 895., 55 557.} 23.491 0.122261
42	newly 3 weeks spec	7600.	7456.27	{2801., 10 584.}	33.5424	0.222608	newly 3 weeks cumul	17 610.	17 510.1	{5068., 28 696.}	43.4061	0.276108	newly 5 weeks spec	7814.	8976.16	{7073., 10 607.}	28.8757	0.14556	newly 5 weeks cumul	33 674.	35 030.5	{16 745., 47 514.}	31.2308	0.186188	newly 56 weeks cumul 56 523. 86 604.9 {80 038., 91 574.} 53.2206 0.184587
43	newly 3 weeks spec	5514.	8290.38	{5967., 9448.}	54.6419	0.192199	newly 3 weeks cumul	14 068.	22 757.5	{12 569., 27 641.}	69.7749	0.235249	newly 5 weeks spec	5382.	7245.26	{4430., 9342.}	41.1385	0.146883	newly 5 weeks cumul	25 003.	38 085.2	{30 074., 42 258.}	55.1505	0.191747	newly 55 weeks cumul 42 797. 61 489.1 {46 846., 71 949.} 44.1513 0.15491
44	newly 3 weeks spec	9897.	12 675.3	{6878., 16 311.}	42.99	0.16597	newly 3 weeks cumul	17 922.	26 282.8	{10 955., 41 221.}	66.5709	0.231144	newly 5 weeks spec	12 427.	12 836.	{8353., 16 248.}	20.947	0.0927528	newly 5 weeks cumul	43 675.	53 315.2	{39 723., 62 129.}	27.3556	0.105435	newly 62 weeks cumul 65 197. 77 994. {73 642., 80 786.} 19.6282 0.0775453
45	newly 3 weeks spec	7988.	7040.04	{4486., 9982.}	28.7262	0.162784	newly 3 weeks cumul	17 198.	24 054.3	{19 821., 28 379.}	47.8784	0.202015	newly 5 weeks spec	9497.	3339.17	{1453., 5661.}	64.8397	0.528224	newly 5 weeks cumul	36 455.	32 496.	{25 800., 40 327.}	17.6444	0.100051	newly 60 weeks cumul 60 722. 38 312.5 {28 384., 49 055.} 36.9051 0.210762
46	newly 3 weeks spec	8753.	6926.67	{1328., 11 434.}	35.7778	0.29518	newly 3 weeks cumul	17 795.	14 031.1	{2330., 25 499.}	41.8936	0.337142	newly 5 weeks spec	10 647.	10 369.5	{5119., 12 353.}	19.4898	0.138098	newly 5 weeks cumul	39 229.	33 515.4	{10 024., 49 383.}	28.2605	0.216641	newly 59 weeks cumul 63 639. 87 806.6 {84 738., 89 973.} 37.9761 0.139691
47	newly 3 weeks spec	6293.	7478.1	{5126., 9702.}	27.6091	0.107364	newly 3 weeks cumul	16 455.	24 702.7	{19 806., 28 145.}	53.135	0.18692	newly 5 weeks spec	6515.	4716.63	{2480., 8062.}	39.4515	0.226246	newly 5 weeks cumul	29 490.	35 540.	{27 118., 42 946.}	25.0794	0.0966446	newly 55 weeks cumul 54 744. 46 370.1 {32 191., 64 026.} 22.3042 0.112773
48	newly 3 weeks spec	8060.	9004.35	{5819., 12 007.}	26.2959	0.111767	newly 3 weeks cumul	18 107.	28 763.2	{24 467., 32 646.}	63.6508	0.222653	newly 5 weeks spec	8793.	4411.19	{2168., 8332.}	51.0641	0.368204	newly 5 weeks cumul	35 919.	39 884.9	{31 542., 46 248.}	17.544	0.0730929	newly 60 weeks cumul 61 312. 46 169.2 {37 910., 56 900.} 24.698 0.130297
49	newly 3 weeks spec	13 841.	15 684.	{6378., 20 339.}	37.7193	0.173268	newly 3 weeks cumul	23 044.	30 718.8	{9005., 45 488.}	61.6923	0.25087	newly 5 weeks spec	12 770.	12 657.6	{6274., 19 864.}	34.177	0.158187	newly 5 weeks cumul	52 454.	59 527.1	{42 343., 66 916.}	21.3218	0.0877056	newly 64 weeks cumul 65 680. 75 945.6 {71 125., 88 330.} 16.7018 0.0665177
50	newly 3 weeks spec	12 684.	16 098.7	{11 728., 18 558.}	32.1726	0.121818	newly 3 weeks cumul	25 589.	40 240.6	{25 065., 48 065.}	64.9159	0.219838	newly 5 weeks spec	9733.	7897.18	{3624., 14 582.}	41.6854	0.223695	newly 5 weeks cumul	48 457.	60 804.6	{53 835., 63 908.}	25.7856	0.0988506	newly 63 weeks cumul 60 105. 68 519.8 {62 892., 73 394.} 14.415 0.0579475
